# Three-dimensional imaging of KNDy neurons in the mammalian brain using optical tissue clearing and multiple-label immunocytochemistry

**DOI:** 10.1038/s41598-018-20563-2

**Published:** 2018-02-02

**Authors:** Aleisha M. Moore, Kathryn A. Lucas, Robert L. Goodman, Lique M. Coolen, Michael N. Lehman

**Affiliations:** 1 Dept. of Neurobiology & Anatomical Sciences, University of Mississippi Medical Center, Jackson, MS USA; 2 Dept. of Physiology and Biophysics, University of Mississippi Medical Center, Jackson, MS USA; 3 Dept. of Physiology and Pharmacology, West Virginia University, Morgantown, West Virginia USA

## Abstract

Kisspeptin/Neurokinin B/Dynorphin (KNDy) neurons of the arcuate nucleus (ARC) play a key role in the regulation of fertility. The ability to detect features of KNDy neurons that are essential for fertility may require three-dimensional (3D) imaging of the complete population. Recently developed protocols for optical tissue clearing permits 3D imaging of neuronal populations in un-sectioned brains. However, these techniques have largely been described in the mouse brain. We report 3D imaging of the KNDy cell population in the whole rat brain and sheep hypothalamus using immunolabelling and modification of a solvent-based clearing protocol, iDISCO. This study expands the use of optical tissue clearing for multiple mammalian models and provides versatile analysis of KNDy neurons across species. Additionally, we detected a small population of previously unreported kisspeptin neurons in the lateral region of the ovine mediobasal hypothalamus, demonstrating the ability of this technique to detect novel features of the kisspeptin system.

## Introduction

Neurons expressing the peptides kisspeptin or neurokinin B (NKB) play a critical role in the regulation of fertility. Mutations that inactivate the genes encoding kisspeptin (*KISS1)*^1^ and its receptor (*GPR54/Kiss1R*)^2,3^, as well as genes encoding NKB (*TAC3)* and its receptor (*TAC3R)*^4^, result in failure to enter puberty and subsequent infertility in humans. Similarly, the deletion of the genes encoding kisspeptin or the receptors for kisspeptin and NKB in the mouse results in subfertility^5,6^ or infertility^7,8^. Within the arcuate nucleus (ARC) there is a unique population of neurons that express both kisspeptin and NKB^9^. In rodent and ovine brains, these neurons are further co-expressed with the endogenous opioid dynorphin A^10–12^ and are now commonly referred to as KNDy neurons. KNDy neurons have become an intense area of focus in neuroendocrinology as regulators of gonadotropin-releasing hormone (GnRH) secretion and steroid hormone feedback. The vast majority of KNDy neurons express steroid hormone receptors^11,13–17^ and are thought to form a reciprocally interconnected network that projects to GnRH neuron cell bodies^11,18–20^ or distal dendrites and terminals^21,22^. Each of the three peptides are hypothesized to play individual roles at both the level of GnRH neurons and reciprocally connected KNDy neurons to form the pulsatile shape of GnRH release^10,23^.

Despite the high degree of homogeneity in the co-expression of KNDy peptides and steroid hormone receptors, it is likely that the KNDy population is composed of functional subunits. In the ewe, there is evidence that KNDy neurons mediate both negative and positive feedback effects of estradiol^24–26^, and a subset of them has been proposed to mediate the latter^26^. However this hypothesis is based on limited data so it remains unclear whether distinct subpopulations of KNDy neurons or the same neurons are responsible for the differential modes of estradiol feedback. Further, KNDy neurons are proposed to influence reproductive capacity by integrating a variety of other cues, including metabolic^27–29^, stress^30^ and seasonal^31^ signals, and to relay the estrogen-mediated control of thermoregulation within the CNS^32^. Taken together, these observations suggest functionally distinct subpopulations of KNDy neurons may regulate GnRH neuron activity and peptide release according to multiple physiological conditions, but little delineation of these subpopulations has occurred.

Previous analyses of the KNDy neural network have relied largely on examination of coronal sections which represent a very limited sampling of the population and may have resulted in some apparent conflicting data on its characteristics (e.g., whether there are more KNDy neurons in the middle or caudal regions of the arcuate in sheep^33^). In contrast, three-dimensional (3D) analysis of the complete intact neuronal circuits has the potential to reveal novel features that are not discernable in sectioned tissue. Therefore, 3D visualization of the complete KNDy cell population and their projections under different physiological states may be necessary to detect anatomical and functional heterogeneity among these cells. Although it is possible to reconstruct 3D circuits from sectioned tissue, this is a time-consuming and intensive process. The last five years have seen an increase in the development and use of optical tissue clearing techniques that permits rapid imaging of fluorescent cell populations in intact organs. In particular, many clearing techniques, such as CLARITY^34^ and CUBIC^35^, are particularly efficient at imaging endogenous fluorescent proteins in transgenic animals. As transgenic technology is largely limited in mammalian species to the mouse, clearing techniques amenable to immunocytochemistry are required for use in other mammalian species. In addition to the mouse, the rat and sheep are the most commonly used models in neuroendocrine research and significant advances in knowledge on KNDy neuroanatomy has been achieved in these species^36^. The sheep has provided a particularly valuable model in which to study neuroendocrine networks given the ability to collect detailed hormonal profiles from both peripheral and portal blood in unanaesthetized animals^37,38^. However, given the size of the rat brain and sheep hypothalamus, it is necessary to adapt immunolabelling and clearing techniques for larger tissue volumes. iDISCO^39,40^, adapted from 3DISCO (three-dimensional imaging of solvent cleared organs)^41^, permits immunolabelling and optical tissue clearing within multiple organs. 3DISCO and iDISCO protocols have been used to visualize cell populations in multiple mouse organs^39,41,42^, the spinal cord of the rat and non-human primate^43^, and human embryos^44,45^. We aimed to further modify iDISCO in order to visualize the complete KNDy population within the rat brain and sheep hypothalamus. To achieve this, we optimized the iDISCO technique for labelling of KNDy peptides and clearing of larger tissue sizes using the rat brain before applying the optimized iDISCO to the sheep hypothalamic block.

## Results

### Whole-mount immunolabelling and optical tissue clearing in the intact rat brain

**Figure 1:**
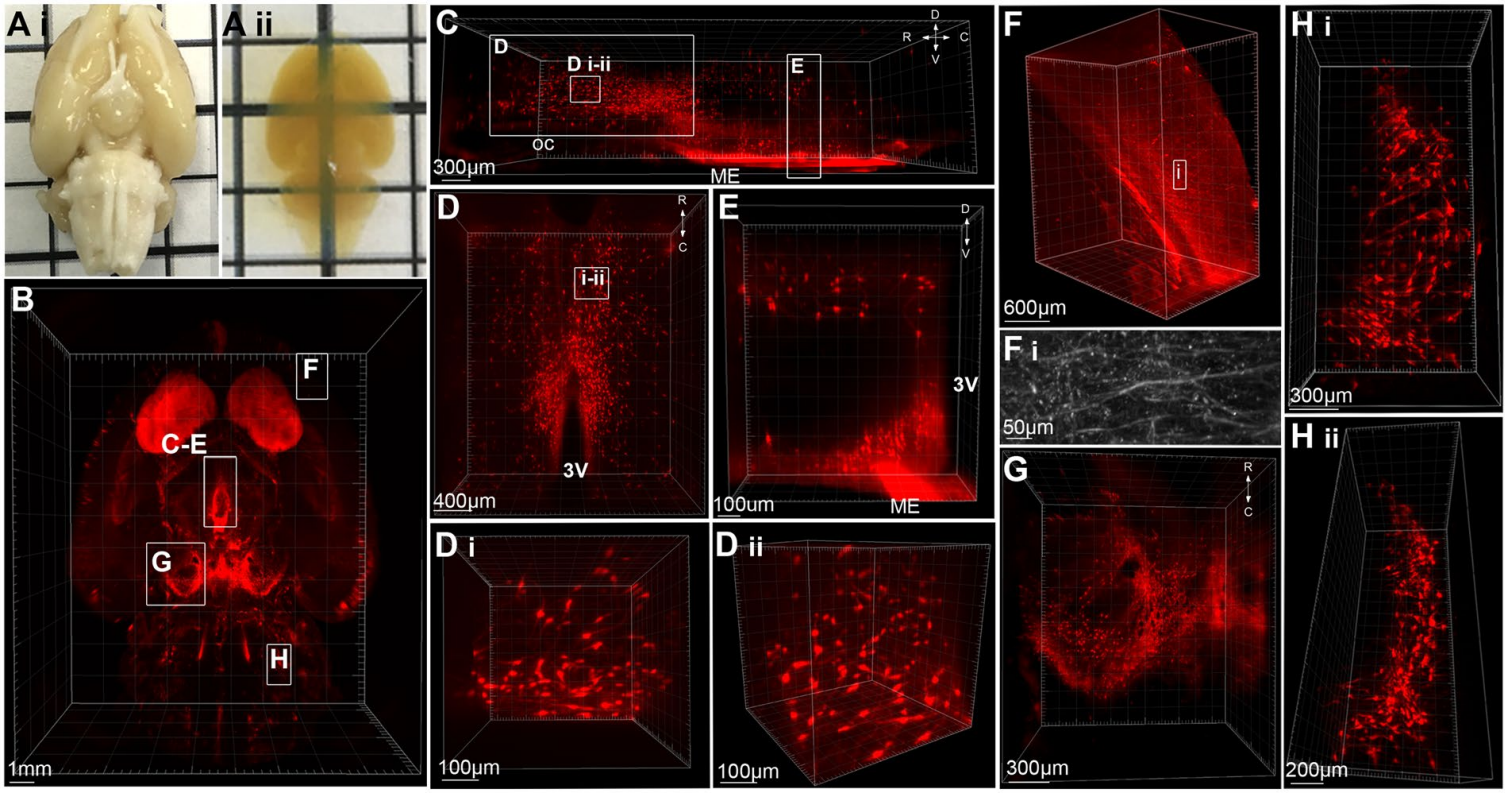
Whole-mount immunolabelling and optical tissue clearing in the rat brain. (**A**) The intact rat brain before (i) after (ii) immunolabelling for tyrosine hydroxylase (TH) and tissue clearing. (**B**) 3D rendering of TH-immunoreactivity (ir) in the rat brain imaged in the horizontal plane and viewed from the ventral surface (z depth = 4 mm). (**C**) Hypothalamic TH-ir neurons viewed in the sagittal plane from −2.6 mm to −4 mm Bregma. Insets (**D**,**E**): (**D**) TH-ir neurons in periventricular nuclei viewed from the dorsal surface with high magnification renderings of neurons in the horizontal plane (**D** i) and rotated to the sagittal (**D** ii) plane. (**E**) TH-ir neurons within a portion of the dorsomedial and arcuate hypothalamic nuclei projected into the coronal plane. (**F**) 3D rendering and a high resolution projected image (**F** i) of TH-ir fibers in the cortex. (**G**) TH-positive neurons within the substantia nigra and ventral tegmental area of the midbrain. (**H**) TH-positive neurons of the A5 group located in the ventrolateral pons viewed in the horizontal plane (i) and rotated into the sagittal plane (ii). R = rostral, C = caudal, D = dorsal, V = ventral, oc = optic chiasm, ME = median eminence, 3 V = third ventricle.

Tyrosine hydroxylase immunolabelling in the intact rat brain was used to optimize iDISCO immunolabelling and clearing for large central nervous system tissue samples (Fig. 1). Post-immunolabelling dehydration of tissue using tetrahydrofuran (THF) was adapted for the larger size of the rat brain, as outlined in Table 1. The length, width and height of whole brain samples shrank by an average of 25.8 ± 0.2%, 28.8 ± 0.03% and 27.7 ± 0.4%, respectively. Complete transparency were achieved (Fig. 1A), and TH-immunoreactivity was detected at a minimum depth of 2.92 mm in samples (4 mm when accounting for shrinkage of tissue) (Fig. 1B). This antibody range permitted imaging of TH-ir cell bodies throughout multiple nuclei in the brain, including the rostral periventricular nucleus of the third ventricle, paraventricular nucleus, and ARC of the hypothalamus (Fig. 1C–E, Video 1), the ventral tegmental area and substantia nigra of the midbrain (Fig. 1G) and the A5 group within the brainstem (Fig. 1H). Further, we achieved sufficient resolution to image TH-ir fibers throughout the brain, including the cortex (Fig. 1F) hypothalamus and ventral striatum. However, finer TH-immunoreactive terminals were not visible within the dorsal striatum (caudate putamen) at a depth of 3.5 mm (5 mm when accounting for shrinkage).

**Table 1:** Tetrahydrofuran-mediated dehydration of rat and sheep tissue for optical tissue clearing.

Tissue	Size	% Tetrahydrofuran in ddH20
Mouse brain (Erturk *et al*. 2012)	1.4 cm × 1 cm × 0.8 cm	50%, 70%, 80%, 100% 1 h, 100% o/n, 100% 1 h
Rat brain block	1.9 cm × 0.9 cm × 0.6 cm	50% o/n, 60%, 70%, 80%, 90%, 96% 1 h, 100% o/n
Whole rat brain	2.5 cm × 1.5 cm × 1.2 cm	50% o/n, 60%, 70%, 80% 2 h, 80% o/n 90%, 96% 100% 2 h, 100% o/n
Sheep hypothalamic block	1.5 cm × 1.5 cm × 1 cm	50% o/n, 60%, 70%, 80%, 2 h, 80% o/n, 90%, 96%, 100%, 100%, 100%, 100% 2 h

### Imaging of the intact arcuate kisspeptin and neurokinin B populations in the rat brain

**Figure 2:**
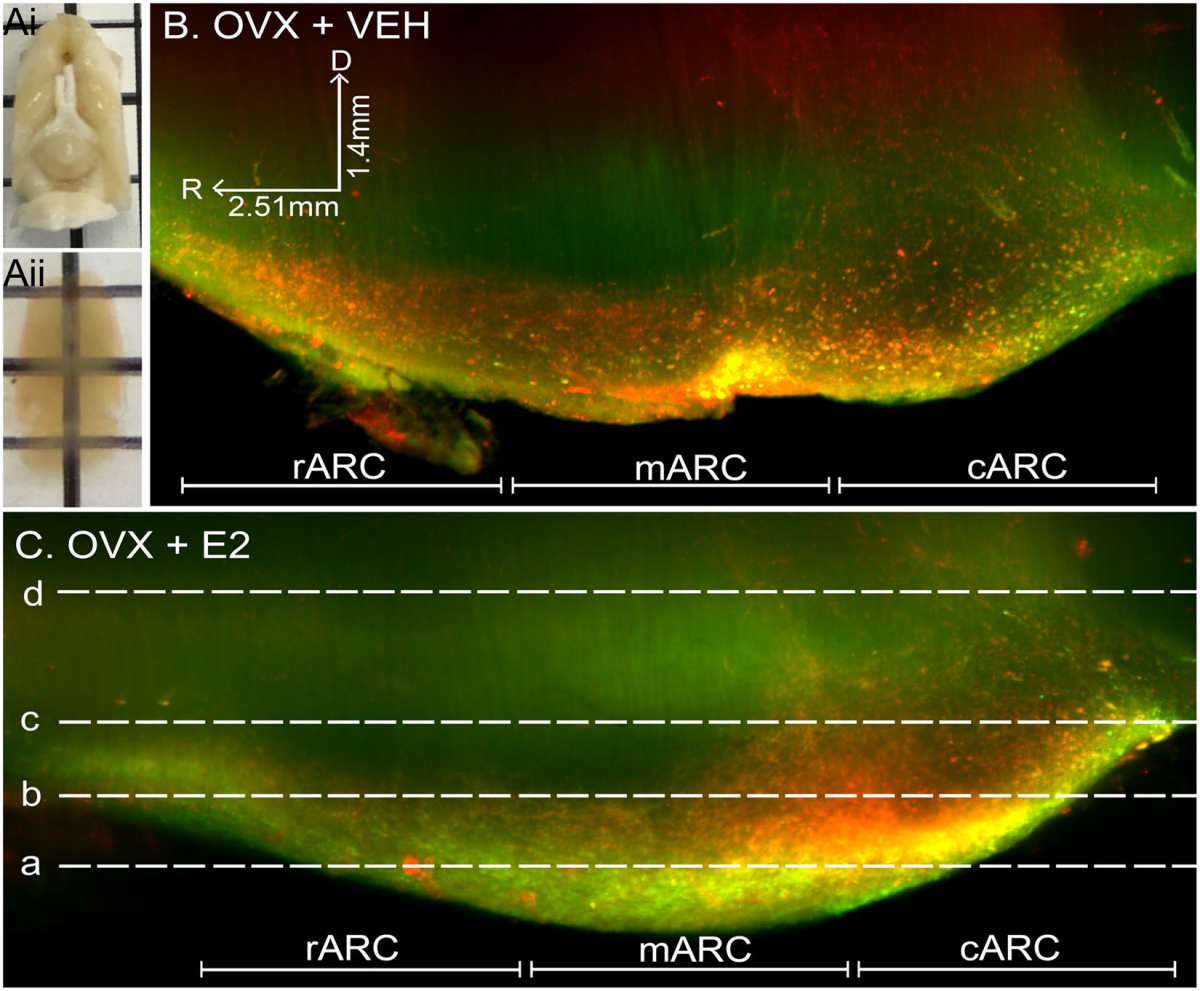
Imaging of the intact KNDy neuron population following immunolabelling and optical tissue clearing. (**A**) Rat brain block before (i) and after (ii) immunolabelling and clearing. (**B**) Projection (optical thickness = 200 µm) of kisspeptin (red) and neurokinin B (green) immunolabelling imaged through the sagittal plane of the arcuate nucleus of ovariectomized rats implanted with either sesame oil vehicle (OVX + VEH, (**B**)) or estradiol (OVX + E2, (**C**)). R = rostral, D = dorsal.

**Figure 3:**
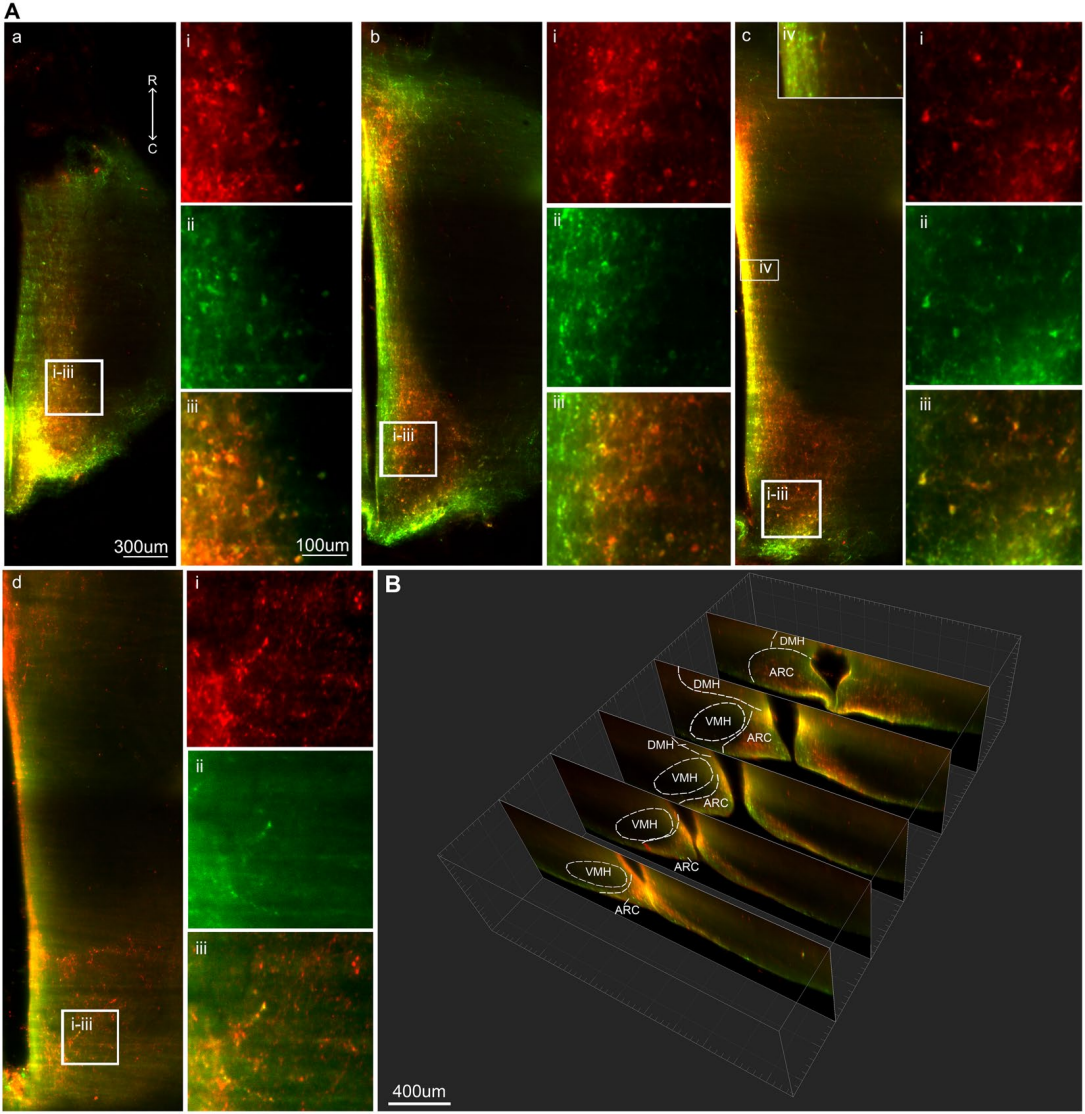
Kisspeptin and neurokinin B immunoreactivity in the cleared rat brain. (**A**) Single optical slices imaged in the horizontal plane with imaging depths of 150 µm (a), 300 µm (b), 450 µm (c) and 750 µm (d) from the ventral surface of the brain. (a–c) ARC kisspeptin and neurokinin B cell bodies. (d) Kisspeptin and NKB fibers in the dorsomedial hypothalamus (DMH). The z-depth of a-d corresponds with the dotted lines in image Fig. 2 (**C**). (i-iii) High magnification images of kisspeptin (i), neurokinin B (ii) and combined immunolabelling (iii) corresponding with insets from (a–d). (iv) Reduced colour saturation reveals dense KNDy fiber projections along the wall of the third ventricle at 450 µm depth from the ventral surface of the ARC. (**B**) Images projected into the coronal plane using IMARIS software showing kisspeptin and neurokinin B immunoreactivity in the rostral to caudal ARC. R = rostral, C = caudal.

**Figure 4:**
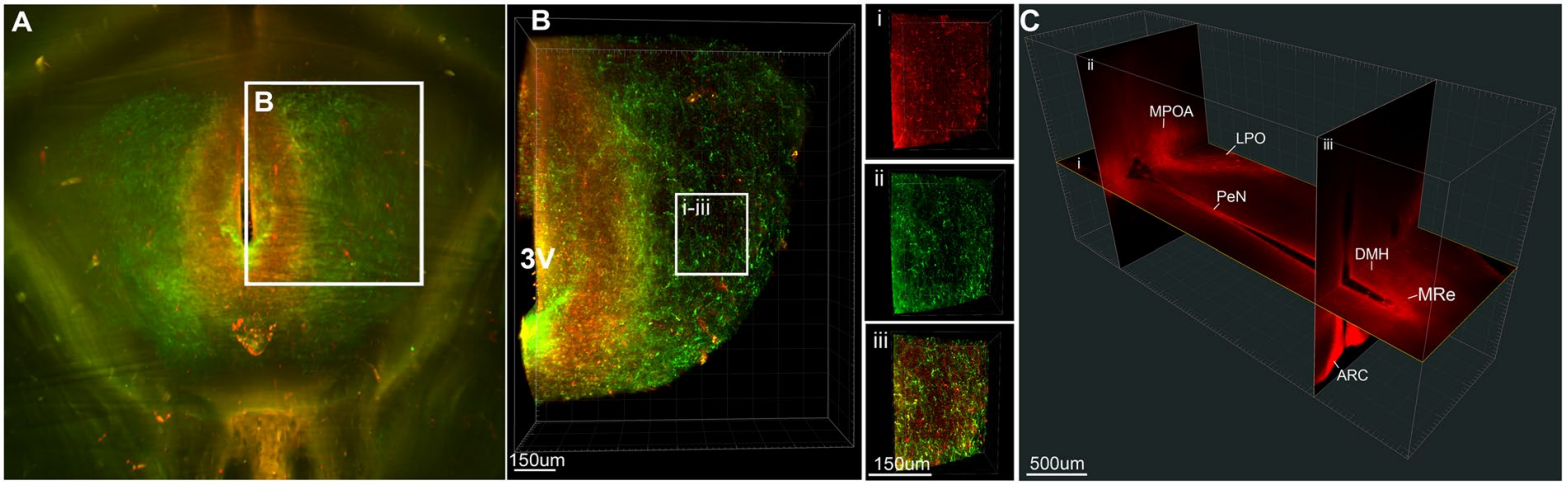
3D rendering of KNDy neuron projections in the rat hypothalamus. Projected image (**A**) and 3D rendering (**B**) of arcuate kisspeptin (red) and neurokinin B (NKB, green) immunolabelling in the arcuate nucleus (ARC) and lateral hypothalamus of an OVX + estradiol rat. (**B** i–iii) Cropped 3D image of arcuate kisspeptin (i), NKB (ii)-positive fibers and the combined labels (iii) from the inset in (**B**). (**C**) Images in the horizontal (i) and coronal (ii-ii) planes rendered from a stack imaged in the sagittal plane containing kisspeptin-ir fibers projecting throughout the hypothalamus, including the medial preoptic area (MPOA), lateral preoptic area (LPO), periventricular nucleus (PeN), dorsomedial hypothalamus (DMH) and the mammillary recess (MRe). 3 V = third ventricle, ARC = arcuate nucleus.

The ability to study KNDy cells without sectioning the population is important for detecting and assessing anatomical features that are not fully discernable in a single plane. Following immunolabelling and optical clearing of rat brains in which the lateral cortices were removed to enhance imaging of midline hypothalamic structures (Fig. 2A), dual-labeled kisspeptin- and NKB-ir cells were observed as a continuum throughout the entire rostral-caudal extent of the ARC of the ovariectomized (OVX) rat treated with oil vehicle (Fig. 2B) or estradiol (Fig. 2C) replacement. Kisspeptin- and NKB-ir cells were also clearly detectable along the complete ventral to dorsal extent of the ARC (Fig. 3), allowing for quantification (Table 2) and 3D rendering of the complete KNDy cell population and projections (Fig. 4). Dense dual-labeled kisspeptin and NKB fibers were imaged projecting from the arcuate nucleus to the lateral hypothalamus (Fig. 4A,B). Few fibers entered the ventromedial hypothalamus (VMH). This is consistent with previous reports^11,46^ and not due to insufficient diffusion of the antibodies as fiber labelling was detected within dorsal hypothalamic structures, including the dorsomedial hypothalamic nucleus (Fig. 3Ad,B). Fiber labelling was also imaged within rostral hypothalamic nuclei, including the medial and lateral preoptic areas and the periventricular nucleus of the third ventricle (Fig. 4C).

**Table 2:** Quantification of kisspeptin- and neurokinin B-positive cell bodies in the arcuate nucleus of ovariectomized (OVX) rats with estradiol replacement (E2) or a vehicle control (VEH).

	Rostral ARC			Middle ARC			Caudal ARC		
	Kp	NKB	Kp + NKB	Kp	NKB	Kp + NKB	Kp	NKB	Kp + NKB
OVX + VEH (n = 4)	259.0 ± 63.1	246.25 ± 54.6	251.0 ± 43.8	761.25 ± 181.0	875.25 ± 210.9	636.5 ± 128.9	1319.8 ± 191.6	1313.0 ± 24.4	1125.5 ± 58
OVX + E2 (n = 4)	157.0 ± 81	175.75 ± 65.3	144.0 ± 72.9	219.75 ± 122.4*	294.25 ± 80.3*	180.75 ± 111.0*	315.5 ± 173*	553.25 ± 200.3*	290.25 ± 161.5*

Kp = Kisspeptin, NKB = neurokinin B.

### Estradiol suppression of kisspeptin and NKB examined in the entire KNDy population using iDISCO

**Figure 5:**
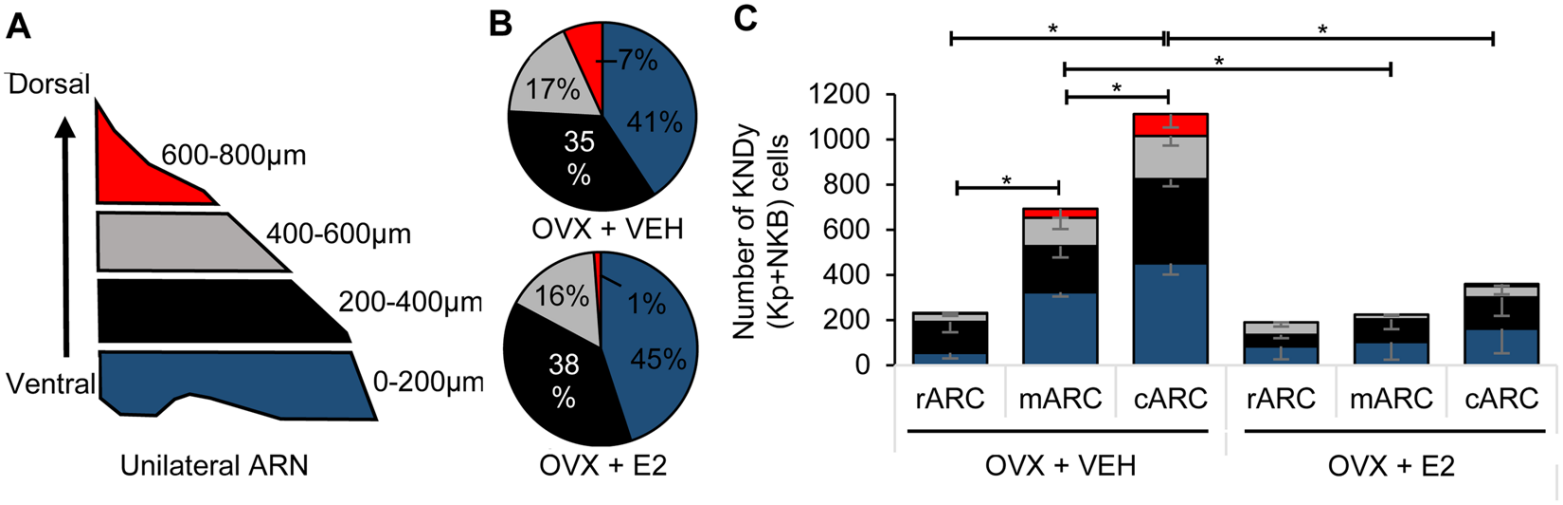
Quantification of the complete KNDy population in vehicle- and estradiol-treated ovariectomized rats. (**A**) Diagram of the dorsal to ventral (d/v) division of the arcuate nucleus (ARC) (coronal plane) by 200 µm intervals. (**F**) The percentage of KNDy cells located in the d/v intervals of the ARC in OVX + VEH and OVX + E2 rats. (**G**) The number of KNDy neurons in the rostral-caudal and ventral-dorsal regions of the ARC in OVX + VEH and OVX + E2 rats. The total number of Kp + NKB (KNDy) cells in the mARN and cARN is significantly decreased in OVX + E2 rats compared to OVX + VEH rats. n = 4/group. *p < 0.05.

Estradiol exerts a profound inhibitory effect on kisspeptin and NKB expression in KNDy cells of adult female rodents^20,47–52^, with OVX animals showing increased numbers of kisspeptin- and NKB-ir cells compared to gonadal-intact or steroid-replaced females. To demonstrate that physiological changes in the arcuate KNDy cell population are detectable using iDISCO, the number of kisspeptin- and NKB-ir cell bodies were compared between OVX + VEH (Fig. 2B) and OVX + E2 (Fig. 2C) rats. As reported previously, the majority of kisspeptin neurons were colocalized with NKB in the rostral ARC (rARC, 86.7 ± 5.9%), middle ARC (mARC, 82.1 ± 4.9%) and caudal ARC (cARC, 85 ± 6.7%) of OVX + VEH rats, and the rARC (91.6 ± 5.9%), mARC (77.5 ± 7.8%) and cARC (92.9 ± 2.7%) of OVX + E2 rats. OVX + E2 rats had significantly reduced kisspeptin, NKB (Table 2) and KNDy (kisspeptin + NKB, Fig. 5C) cell numbers in the mARC and cARC compared with OVX + VEH rats, but this did not reach significance within the rARC. The number of kisspeptin and NKB-ir colocalized cells was significantly different between the rostral, middle and caudal arcuate of OVX + VEH rats (Table 2), and this significance was lost in OVX + E2 animals (Fig. 5C). The distribution of KNDy cells was also mapped from the ventral to dorsal plane through the ARC (Fig. 5A), demonstrating that the highest density of KNDy cells were present in the most ventral regions of the ARC (Fig. 5B). This distribution was not significantly different between OVX + VEH and OVX + E2 rats.

### Kisspeptin and GnRH immunolabelling after optical tissue clearing of the intact ovine hypothalamus

**Figure 6:**
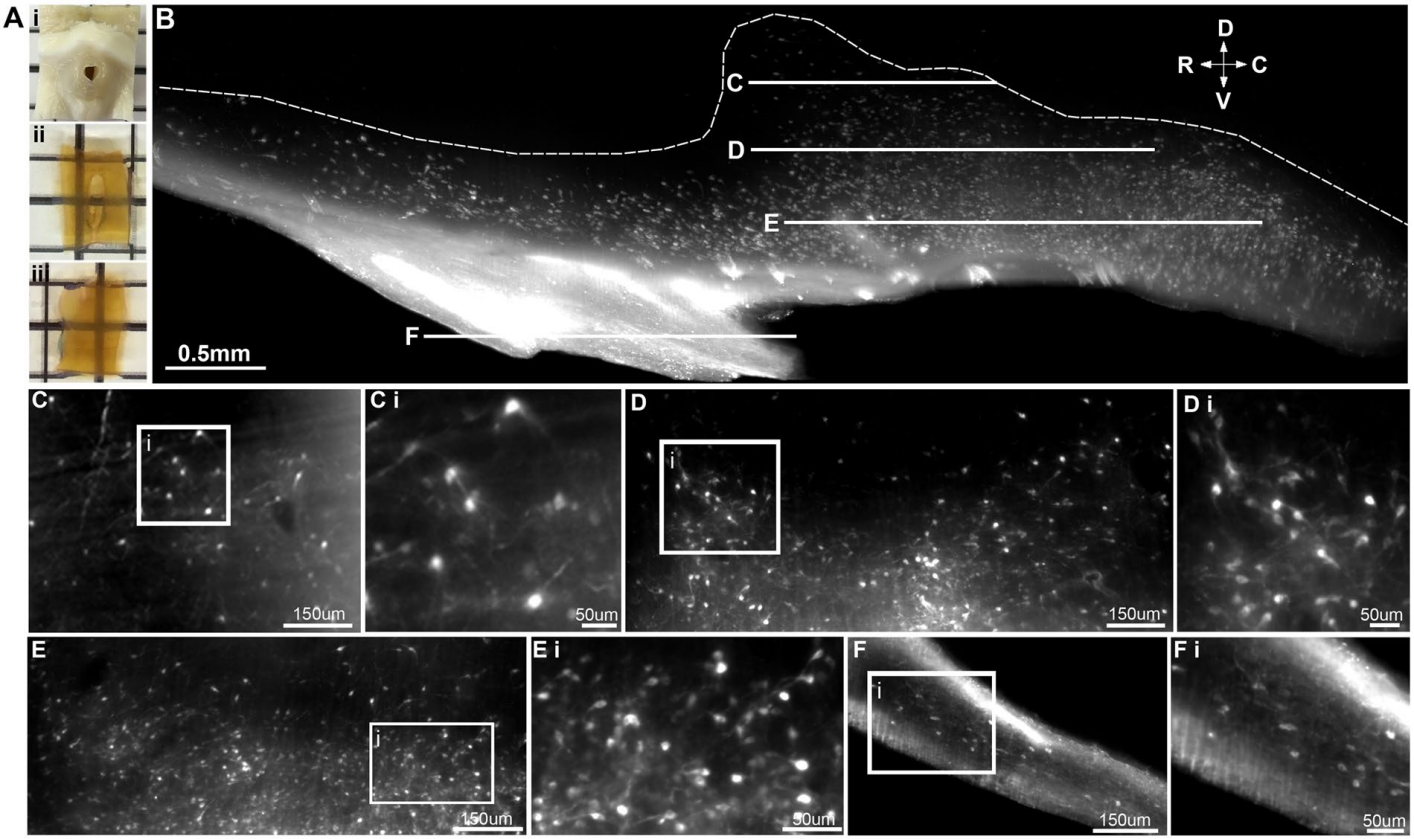
Immunolabelling and optical tissue clearing in the ovine hypothalamus. (**A**) Ovine hypothalamic block before (i) and after immunolabelling and optical tissue clearing as viewed in the horizontal (ii) and sagittal (iii) plane. (**B**) Projected image of kisspeptin neurons in the ovine arcuate nucleus (ARC) in the sagittal plane (optical thickness = 250 µm). Dotted outline represents the dorsal border of the ARC. (**C**–**F**) Single optical slices (4 µm optical thickness) through the ARC imaged in the horizontal plane. The z-depth of each image corresponds with lines (**C**–**F**) in image (**B**). (**C**–**F**) (i) High magnification images of kisspeptin neurons corresponding with the insets in C-F. D = dorsal, V = ventral, R = rostral, C = caudal.

**Figure 7:**
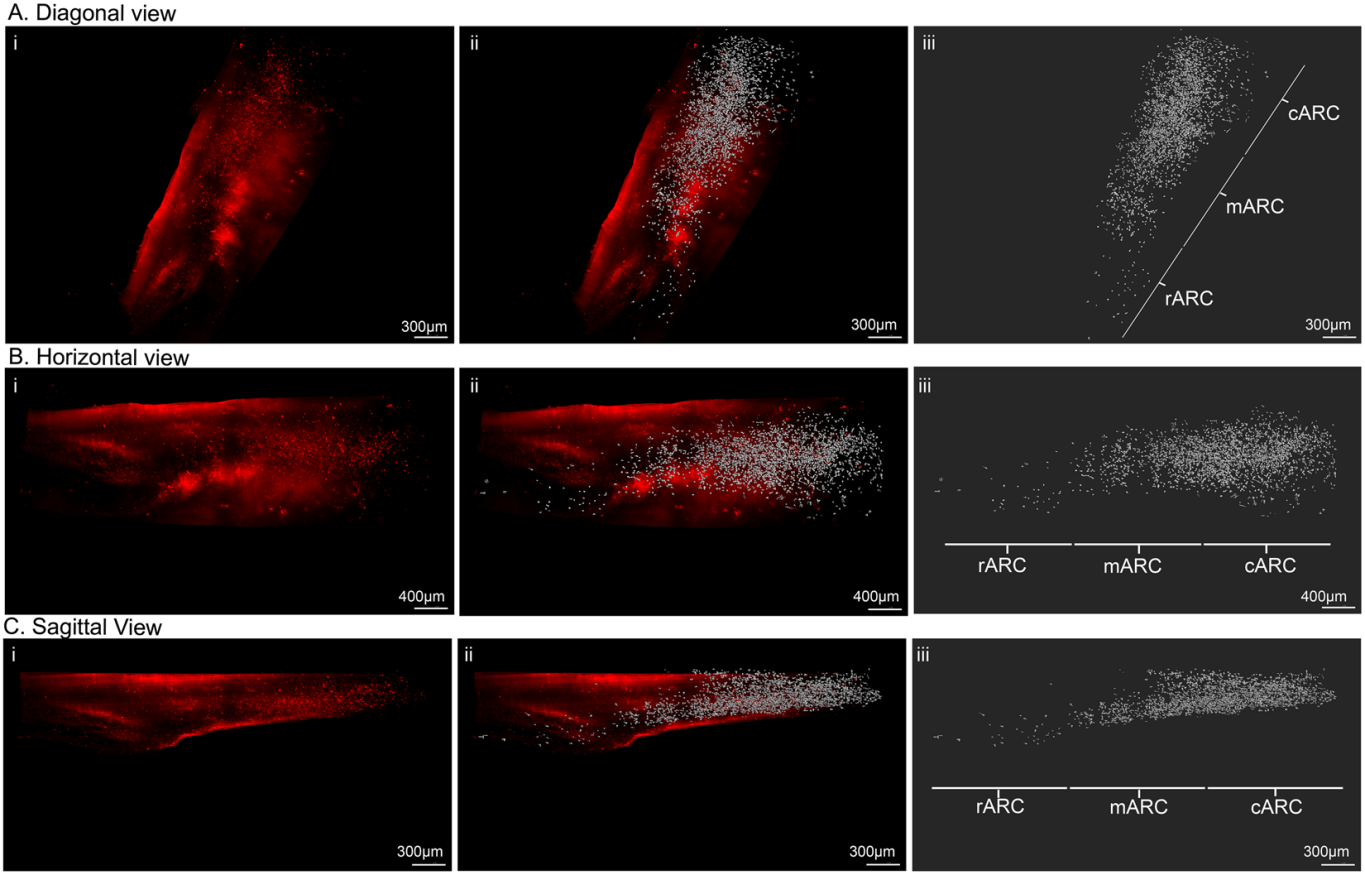
Three-dimensional visualization of kisspeptin neurons in the ovine arcuate nucleus. Three-dimensional (3D) rendering of kisspeptin cell bodies in the intact unilateral arcuate nucleus as viewed from the diagonal (**A**), horizontal (**B**) and sagittal (**C**) planes (i). 3D rendering of kisspeptin immunoreactivity (i) with isosurface visualization of kisspeptin cells (ii) and the isosurface alone (iii). The rostral (rARC), middle (mARC) and caudal (cARC) regions of the ARC are outlined in iii.

**Figure 8:**
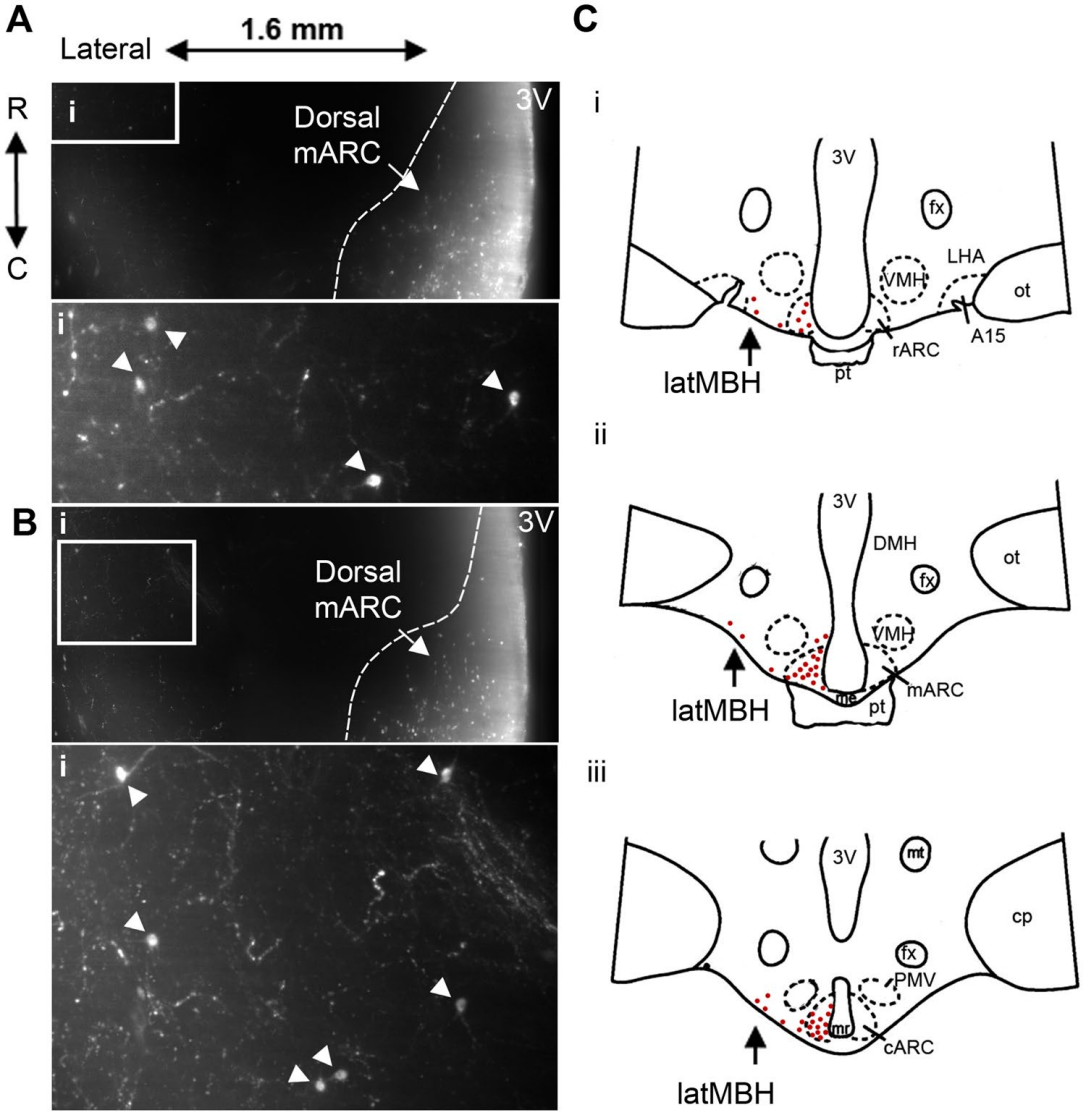
Kisspeptin neurons in the lateral mediobasal hypothalamus as identified via volumetric imaging. (**A**,**B**) Arcuate and lateral mediobasal hypothalamic kisspeptin neurons within single optical slices (4 µm optical thickness) in the horizontal plane. Dotted lines represent the border of the dorsal middle arcuate nucleus (mARC). The depth from the ventral surface in A = 0.62 mm and B = 0.84 mm. (i) Insets from corresponding images with arrows indicating kisspeptin cell bodies in the lateral mediobasal hypothalamus. (**C**) Representative diagrams of the ovine hypothalamus in the coronal plane containing the rostral (i), middle (ii) and caudal (iii) regions of the ARC. The relative density of kisspeptin cell bodies within the MBH are displayed as red dots. Kisspeptin cell bodies in the lateral region of the MBH (latMBH) are indicated by black arrows.

Hypothalamic blocks were immunolabelled for kisspeptin and rendered transparent using an adapted iDISCO clearing protocol (Table 1, Fig. 6A). Immunoreactive kisspeptin cell bodies were detected along the entire rostral to caudal extent (Fig. 6B) and ventral to dorsal extent of the ARC (Fig. 6C–F), allowing 3D reconstruction of the arcuate kisspeptin neuronal population (Fig. 7, Video 2). As a demonstration that this technique is adaptable for use with multiple immunolabelling in the sheep as in the rat brain, we processed sheep hypothalamic blocks for dual-labeling of both kisspeptin and GnRH immunolabelling (Supplementary Figure 1). GnRH fibers could be viewed projecting through the arcuate kisspeptin population before terminating in the median eminence. In addition, a small number of scattered kisspeptin-ir cell bodies were detected within the lateral region of the mediobasal hypothalamus ventral to the fornix (Fig. 8A,B). These scattered cell bodies were located up to 1 mm from the ventral surface of the brain. The rostral to caudal extent of this previously unreported population lay in parallel with the rostral to caudal extent of the ARC (Fig. 8C).

## Discussion

We report here the application of iDISCO, a solvent-based optical tissue clearing protocol commonly used for imaging of intact cellular structures, to study the arcuate KNDy neuron population in the rat and sheep brain. To our knowledge, we provide the first 3D visualization of the arcuate KNDy population in any species using a dual-label approach with kisspeptin and NKB antibodies, and, the first use of iDISCO clearing and immunolabeling in sheep tissue. A recent study using CLARITY-mediated clearing of the intact mouse brain enabled visualization of kisspeptin neurons in a transgenic Kiss1-Cre mouse line crossed with a tdTomato reporter^53^. This provided a complete view of endogenously fluorescent kisspeptin neurons, and confocal imaging of cleared thick brain slices permitted detailed tracing of kisspeptin projections throughout the hypothalamus. Here, the use of immunolabelling and clearing with iDISCO to study kisspeptin neurons in the rat and sheep expands this capability into other non-transgenic species, permits the study of multiple proteins at once, and avoids the ectopic or developmental expression of fluorescent reporter proteins that are common within transgenic lines.

In addition to visualization of the KNDy network, we also report clearing of the intact rat brain through modification of THF-mediated dehydration. The rat is a widely used model in neuroscience, and is particularly useful in behavioral, pharmacological and physiological studies. Despite this, there are limited reports of clearing and 3D imaging of the adult rat brain. One study utilized the FluoClearBABB protocol, based on dehydration with tert-butanol^54^, to render the brain of a transgenic Thy1-GFP rat transparent for subsequent light sheet imaging of the GFP reporter protein^55^. As there are limited transgenic rat models available, the current report describes a method that utilizes immunolabelling and can be applied to multiple cell populations within the central nervous system of the rat. We achieved antibody penetration through the majority of the intact rat brain with tyrosine hydroxylase labelling. However, TH-immunoreactive terminals could not be imaged in the dorsal striatum at the midline of the brain. This may be caused by limitations of the selected antibody concentration, the resolution of the lightsheet microscope, or the working distance of the objective. It is therefore recommended to trim tissues to either the biological region of interest or to single hemispheres to optimize labelling and imaging of midline structures.

The ability to observe the complete KNDy neuron population in cleared tissue overcomes the disadvantages of traditional sectioning techniques, such as limited sampling from subpopulations across the ARC and sectioning fiber tracts. We achieved imaging of the complete continuum of kisspeptin and NKB neurons throughout the entire rostral to caudal extent of the ARC. The suppression of kisspeptin and NKB expression by estradiol in the middle and caudal regions of the rat ARC was quantifiable, indicating that the adapted iDISCO protocol can be used to detect physiological changes in the complete KNDy network. Estradiol-mediated suppression of kisspeptin mRNA and protein^20,47^ and NKB mRNA^47–50^ and protein^51,52^ has been documented across species. However, one study using immunolabelling in the rat reported a significant increase in the number of NKB-positive cells in the caudal ARC with estradiol treatment^56^. It is possible that the opposing results reflect different concentrations of E2 used, as E2 replacement also failed to reduce ARC kisspeptin cell numbers following OVX in the aforementioned study^56^.

Three-dimensional rendering of the arcuate kisspeptin population in the ewe demonstrated the sparse number of kisspeptin neurons in the rostral ARC when compared to the middle and caudal regions, consistent with previous reports^13^. In the middle and caudal ARC, the number of kisspeptin neurons is significantly higher in females compared to males^57^, suggesting that KNDy neurons controlling the sexually differentiated pattern of GnRH release are located here. Additionally, in an ovine model of PCOS which exhibits increased GnRH/LH pulse frequency and an abolished GnRH/LH surge, NKB and dynorphin expression is reduced in the middle and caudal ARC, further supporting that KNDy neurons in this region are important regulators of GnRH release^57^. Notably, in 3D projections, the number of kisspeptin neurons appeared greater in the caudal ARC compared to the middle ARC. This was likely underestimated in traditional sectioning approaches and highlights the possible functional separation of the caudal portion of the nucleus from the middle and rostral divisions. This functional separation is supported by a recent report using ICV delivery of antagonists to the serotonin receptor (SSTR2) in the ewe, which activated kisspeptin neurons specifically in the caudal region of the ARC^58^. In future experiments, the complete kisspeptin population can be assessed in 3D under differing physiological and pathological conditions. For example, markers of activation, such as Fos, can be used to detect activated subpopulations of kisspeptin neurons during differential modes of GnRH release. Although this is achievable with the current resolution of the lightsheet microscope, the investigation of fine neuroanatomical details (such as dendritic spines) and subcellular markers (such as G-protein coupled receptors to mark the release of endogenous neurotransmitters) will require future technological advances to improve the lateral resolution of the lightsheet microscope, which is currently limited to 1 µm.

The ability to image large areas of the intact sheep hypothalamus revealed a previously unreported population of kisspeptin neurons. Besides the ARC, kisspeptin neurons in the ovine MBH and surrounding regions had previously been reported within the DMH and VMH^59^. Here, in addition to these previously identified populations, we detected a scattered, small number of kisspeptin-positive neurons within the lateral region of the mediobasal hypothalamus. The functional role of this kisspeptin population is not yet known, although there are reports of scattered GnRH neurons located within this brain region^60,61^.

Dehydration of tissue with THF during the clearing process shrunk tissue volume by an average of 27.4%. This is in line with what has previously been reported using THF-mediated clearing, under which mouse brain samples have shrunk up to 50%^62^. Tissue shrinkage in clearing protocols has recently been taken advantage of for more rapid lightsheet imaging of large areas, such as described in the “ultimate DISCO” (uDISCO) protocol, which shrinks tissue by 65%^63^. Although this is an advantage for larger tissue samples such as the sheep hypothalamus, it is possible that distortion of the tissue can occur. The recently published iDISCO + protocol is reported to reduce shrinkage to 10% in cleared mouse brain samples by replacing post-immunolabelling dehydration of brain tissue by THF with methanol^64^. However, in our hands, the intact rat brain shrank by 26–27% with iDISCO+, indicating that shrinkage still occurs in larger tissue samples with methanol-based dehydration (data not shown).

Although the iDISCO system offers an important added 3D view of the complete distribution of a neuronal population there are a number of considerations when applying to larger animal models, which can be expensive and time consuming to produce. First, autofluorescent labelling of blood vessels were more pronounced in ovine tissue when compared to the rodent brain, although this did not prevent labelling of kisspeptin neurons. Second, not all antibodies are compatible with solvent-based tissue clearing. Importantly, traditional sectioning techniques provide multiple series for experimental analysis, whereas using a complete brain limits the number of antibodies that can be visualized per animal, and thus, the number of experimental data points. Therefore, iDISCO is likely to be used in conjunction with traditional sectioning techniques, but does not entirely replace 2D histological analysis.

In conclusion, we report adaptation of the iDISCO technique for high resolution imaging of the complete KNDy neuron population in the transparent rat and sheep brain. This technique permits versatile analysis of the KNDy population, including the extraction of detailed 3D information regarding cell numbers and fiber distribution. In addition, the ability to render the intact rat brain and sheep hypothalamus transparent provides the broad opportunity to interrogate a variety of cell populations throughout the central nervous system of species other than mice that are commonly used in neuroscience, including large animal models.

## Materials and Methods

### Animals

Adult female Sprague Dawley rats (Charles River) were housed in separate rooms, in same sex-pairs in standard Plexiglass cages with ad libitum access to food and water. Animals were maintained in temperature and humidity controlled rooms on a 12-hour dark/light cycle with lights off at 0900 hr. Adult black-faced ewes of mixed breeding were housed in an open barn. Ewes were given open access to water and fed once daily with a maintenance regimen of silage. Animals were moved into an indoor facility 3–5 days before surgery in which the duration of artificial lighting was adjusted to match that outdoors. They were fed a diet of alfalfa cubes, and had free access to water and minerals.

### Ovariectomy and tissue preparation

Female rats were bilaterally ovariectomized from the ventral surface and implanted with sc capsules (Dow Corning tubing, 1.98 mm internal diameter, 30 mm length), prepared as described in^65^, containing either sesame oil vehicle (n = 4) or 100 µg/mL of 17β estradiol-benzoate dissolved in sesame oil (n = 4)^66^. Eight days following OVX, rats were deeply anesthetized using ip injection of sodium pentobarbital (270 mg/kg, Sigma-Aldrich) and perfused intracardially with 10 mL of 0.9% saline, followed by 500 mL of 4% paraformaldehyde (PFA) in 0.1M phosphate buffer (PB). Rats were decapitated post-perfusion, brains were extracted from the skull and post-fixed in 4% PFA overnight at 4 C. The lateral cortices were removed using vertical cuts lateral to the amygdala in OVX rats (Fig. 3A) so to enhance imaging of midline hypothalamic structures with horizontal lightsheet microscopy. Ovary-intact rats (n = 4) were anesthetized and perfused as described above, and brains were kept intact. Rat brains were kept in 0.1 M PB (pH 7.3) with 0.01% sodium azide until immunolabelling and optical tissue clearing protocols were performed.

Ewes were bilaterally ovariectomized by midventral laparotomy under isoflurane anesthesia (2–4%) using sterile techniques^67^ and treated with dexamethasone and penicillin pre- and post-operatively. Daily analgesia (Banamine, Phoenix Pharmaceutical, St Joseph, MO, USA; 125 mg/sheep) was given starting at time of anesthesia through to 5 days post-surgery. Two weeks following surgery, OVX ewes were euthanized with iv sodium pentobarbital (2–3 g; Sigma Aldrich) and decapitated. The heads were perfused through both internal carotid arteries with 6L of 4% PFA in 0.1M PB (pH 7.3) mixed with 0.1% sodium nitrite and 10 U/mL heparin. Whole brains were removed from the skull post perfusion and placed into 0.1M PB with 0.01% sodium azide. Hypothalamic tissue blocks measuring approximately 1.5 cm (length) x 1.5 cm (width) x 1 cm (height) were prepared (n = 4). Meninges were removed from the tissue blocks, and the majority of the infundibular stalk was carefully removed for optimal visualization of the ARC under the lightsheet microscope. All procedures were approved by the University of Mississippi Medical Center Animal Care and Use Committee (rats) and the West Virginia University Animal Care and Use Committee (sheep) and followed National Institutes of Health guidelines for animal research.

### Antibodies

The primary antibodies used in these protocols were polyclonal mouse anti-tyrosine hydroxylase (1:1000, Sigma, RRID: AB_477569), polyclonal rabbit anti-kisspeptin (1:250, A566, RRID: AB_2622231, kindly gifted by A. Caraty), polyclonal guinea pig anti-proNKB (1:250, IS-3/63, kindly gifted by P. Ciofi) and polyclonal mouse anti-GnRH (Millipore, 1:150, MAB5456). Lower concentrations of tyrosine hydroxylase (1:5000), kisspeptin and NKB (1:500, 1:1000) primary antibodies detected few cell bodies or fibers in cleared brains from OVX rats (data not shown). The secondary antibodies used were Alexa Fluor goat anti-rabbit 647, Alexa Fluor goat anti-mouse 647, Alexa Fluor goat anti-mouse 555, Alexa Fluor goat anti-guinea pig 555 and Alexa Fluor goat anti-mouse 555 (Invitrogen, 1:100). All antibodies have been previously characterized in rats and sheep and validated for specificity with target peptides^12,13,68,69^.

### Immunolabelling-enabled 3D imaging of solvent cleared organs (iDISCO)

As outlined in^39^, rat and sheep brain tissue was dehydrated in methanol, bleached with hydrogen peroxide, rehydrated, and blocked using normal goat serum. The brain tissue was incubated with shaking in blocking serum, primary antibodies and secondary antibodies for 7 days at 37 C at each step to permit diffusion through the large tissue samples. Brains from ovary-intact rats (n = 4) were immunolabelled using mouse anti-TH serum followed by Alexa Fluor goat anti-mouse 647 secondary antibody. Brains from OVX + VEH and OVX + E2 rats were dual-labeled using rabbit anti-kisspeptin and guinea pig anti-NKB, which were detected using Alexa Fluor goat anti-rabbit 647 and Alexa Fluor goat anti-guinea pig 555 secondary antibodies, respectively. Sheep hypothalamic blocks were immunolabelled using either rabbit anti-kisspeptin or both rabbit anti-kisspeptin and mouse anti-GnRH antibodies, followed by goat anti-rabbit 647 or goat anti-mouse 555 secondary antibodies, respectively.

Post-immunolabelling dehydration of tissue using tetrahydrofuran (THF) was adapted for the larger size of the rat brain and sheep hypothalamic block, as outlined in Table 1. Following dehydration, brains were rotated in dichloromethane for a maximum of 2 hours before being rendered transparent using incubation in dibenzyl ether (DBE). Transparent tissue was submerged in a chamber containing DBE and imaged in the horizontal or sagittal plane using a bidirectional light-sheet microscope (LaVison BioTec) with a 2×/0.5NA objective (MVPLAPO Olympus). Stacks of TIFF images were collected using a sCMOS camera (Andor Neo) with ImSpectorPro software (LaVision BioTec) at either 0.63×, 0.8×, 1×, 1.25×, 1.6×, 2×, 2.5×, 3.2×, 4×, 5× or 6.3× magnification with a 4 μm optical interval.

### Image processing

Mosaic stacks of 16-bit TIFF images were stitched together using Fiji software to generate a single z-stack containing the imaged areas. To quantify the number of kisspeptin and NKB labelled neurons, users were blinded to experimental treatment and counts confirmed by a second user. In TIFF z-stacks, the rostral, middle and caudal regions of the ARC were outlined using the ‘regions of interest’ tool in Fiji. The ‘multi-point’ tool was used to count the number of kisspeptin or NKB positive cell bodies within each arcuate region in 200 µm segments from the ventral surface up to 1 mm. To count the number of neurons containing both peptides, the users switched between channels. Three-dimensional volume files and movie files were generated using IMARIS software (Bitplane). Stacks were converted to Imaris files (.ims) and 3D projections of z-stack images were generated using the volume rendering function. In ovine tissue, stacks of images measuring mm 1.14 mm (middle to lateral) by 3.90 mm (rostral to caudal) by 1.74 mm (ventral to dorsal) containing the ARC were projected in 3D. Regions of interest in 3D reconstructions were isolated using the 3D crop tool. In the ovine tissue, large autofluorescent blood vessels lateral and medial to the kisspeptin population in the caudal ARC, and a small region of autofluorescence along the third ventricle wall dorsal to the ARC, was removed using the surface tool and the mask function (Supplementary Figure 2). Kisspeptin cells in the ovine ARC (excluding the median eminence and infundibular stalk) were reconstructed using IMARIS segmentation tools. Three-dimensional images and movies were generated using the ‘snapshot’ and ‘animation’ tools. The ovine ARC was divided into three 1.25 mm lengths during animation to define the rostral, middle and caudal regions of the nucleus. Movies were edited using Apple iMovie software.

### Statistics

The number of single labelled kisspeptin and NKB cells and the number of KNDy (Kisspeptin + NKB co-labeled) cells were compared between OVX + VEH and OVX + E2 treated rats in the rostral, middle and caudal ARC using two-way ANOVA with Tukey post-tests (Sigma Plot). All data are expressed as mean + SEM. A p value of <0.05 was considered statistically significant.

### Data availability statement

The datasets generated and analyzed during the current study are available from the corresponding author upon request.

## Electronic supplementary material

Supplementary information

Video 1—Animated 3D rendering of hypothalamic tyrosine hydroxylase neurons

Video 2—Animated 3D rendering of arcuate kisspeptin neurons in the ovine hypothalamus

## References

[1] Topaloglu AK (2012). Inactivating KISS1 mutation and hypogonadotropic hypogonadism. New England Journal of Medicine.

[2] de Roux N (2003). Hypogonadotropic hypogonadism due to loss of function of the KiSS1-derived peptide receptor GPR54. Proceedings of the National Academy of Sciences.

[3] Seminara SB (2003). The GPR54 gene as a regulator of puberty. New England Journal of Medicine.

[4] Topaloglu AK (2009). TAC3 and TACR3 mutations in familial hypogonadotropic hypogonadism reveal a key role for Neurokinin B in the central control of reproduction. Nature genetics.

[5] Lapatto R (2007). Kiss1−/− mice exhibit more variable hypogonadism than Gpr54−/− mice. Endocrinology.

[6] Yang JJ, Caligioni CS, Chan Y-M, Seminara SB (2012). Uncovering novel reproductive defects in neurokinin B receptor null mice: closing the gap between mice and men. Endocrinology.

[7] de Tassigny, X. d. A. *et al*. Hypogonadotropic hypogonadism in mice lacking a functional Kiss1 gene. *Proceedings of the National Academy of Sciences* **104**, 10714–10719 (2007). 10.1073/pnas.0704114104 PMC1965578 17563351

[8] Funes S (2003). The KiSS-1 receptor GPR54 is essential for the development of the murine reproductive system. Biochemical and biophysical research communications.

[9] Ramaswamy S (2010). Neurokinin B stimulates GnRH release in the male monkey (Macaca mulatta) and is colocalized with kisspeptin in the arcuate nucleus. Endocrinology.

[10] Navarro VM (2009). Regulation of gonadotropin-releasing hormone secretion by kisspeptin/dynorphin/neurokinin B neurons in the arcuate nucleus of the mouse. The Journal of neuroscience: the official journal of the Society for Neuroscience.

[11] Burke MC, Letts PA, Krajewski SJ, Rance NE (2006). Coexpression of dynorphin and neurokinin B immunoreactivity in the rat hypothalamus: morphologic evidence of interrelated function within the arcuate nucleus. Journal of Comparative Neurology.

[12] Goodman RL (2007). Kisspeptin neurons in the arcuate nucleus of the ewe express both dynorphin A and neurokinin B. Endocrinology.

[13] Franceschini I (2006). Kisspeptin immunoreactive cells of the ovine preoptic area and arcuate nucleus co-express estrogen receptor alpha. Neuroscience letters.

[14] Goubillon ML (2000). Identification of neurokinin B-expressing neurons as an highly estrogen-receptive, sexually dimorphic cell group in the ovine arcuate nucleus. Endocrinology.

[15] Smith JT, Cunningham MJ, Rissman EF, Clifton DK, Steiner RA (2005). Regulation of Kiss1 gene expression in the brain of the female mouse. Endocrinology.

[16] Ciofi P, Krause JE, Prins GS, Mazzuca M (1994). Presence of nuclear androgen receptor-like immunoreactivity in neurokinin B-containing neurons of the hypothalamic arcuate nucleus of the adult male rat. Neurosci Lett.

[17] Foradori CD (2002). Colocalization of progesterone receptors in parvicellular dynorphin neurons of the ovine preoptic area and hypothalamus. Endocrinology.

[18] Foradori CD, Amstalden M, Goodman RL, Lehman MN (2006). Colocalisation of dynorphin a and neurokinin B immunoreactivity in the arcuate nucleus and median eminence of the sheep. J Neuroendocrinol.

[19] Merkley, C. M., Coolen, L. M., Goodman, R. L. & Lehman, M. N. Evidence for changes in numbers of synaptic inputs onto KNDy and GnRH neurones during the preovulatory LH surge in the ewe. *Journal of neuroendocrinology* (2015). 10.1111/jne.12293 PMC4809364 25976424

[20] Navarro V (2011). Regulation of NKB pathways and their roles in the control of Kiss1 neurons in the arcuate nucleus of the male mouse. Endocrinology.

[21] Hoong Yip, S., Boehm, U., Herbison, A. E. & Campbell, R. E. Conditional viral tract-tracing delineates the projections of the distinct kisspeptin neuron populations to gonadotropin-releasing hormone (GnRH) neurons in the mouse. *Endocrinology*, en. 2015–1131 (2015). 10.1210/en.2015-1131 25856430

[22] True C, Kirigiti M, Ciofi P, Grove KL, Smith MS (2011). Characterisation of arcuate nucleus kisspeptin/neurokinin B neuronal projections and regulation during lactation in the rat. J Neuroendocrinol.

[23] Lehman MN, Coolen LM, Goodman RL (2010). Minireview: kisspeptin/neurokinin B/dynorphin (KNDy) cells of the arcuate nucleus: a central node in the control of gonadotropin-releasing hormone secretion. Endocrinology.

[24] Merkley CM (2012). KNDy (kisspeptin/neurokinin B/dynorphin) neurons are activated during both pulsatile and surge secretion of LH in the ewe. Endocrinology.

[25] Smith JT, Li Q, Pereira A, Clarke IJ (2009). Kisspeptin Neurons in the Ovine Arcuate Nucleus and Preoptic Area Are Involved in the Preovulatory Luteinizing Hormone Surge. Endocrinology.

[26] Estrada KM, Clay CM, Pompolo S, Smith JT, Clarke IJ (2006). Elevated KiSS-1 expression in the arcuate nucleus prior to the cyclic preovulatory gonadotrophin-releasing hormone/lutenising hormone surge in the ewe suggests a stimulatory role for kisspeptin in oestrogen-positive feedback. Journal of Neuroendocrinology.

[27] Cernea M, Phillips R, Padmanabhan V, Coolen LM, Lehman MN (2016). Prenatal testosterone exposure decreases colocalization of insulin receptors in kisspeptin/neurokinin B/dynorphin and agouti‐related peptide neurons of the adult ewe. European Journal of Neuroscience.

[28] Backholer K (2010). Kisspeptin cells in the ewe brain respond to leptin and communicate with neuropeptide Y and proopiomelanocortin cells. Endocrinology.

[29] Castellano JM, Bentsen AH, Mikkelsen JD, Tena-Sempere M (2010). Kisspeptins: bridging energy homeostasis and reproduction. Brain Res.

[30] Ralph CR, Lehman MN, Goodman RL, Tilbrook AJ (2016). Impact of psychosocial stress on gonadotrophins and sexual behaviour in females: role for cortisol?. Reproduction (Cambridge, England).

[31] Weems PW, Goodman RL, Lehman MN (2015). Neural mechanisms controlling seasonal reproduction: principles derived from the sheep model and its comparison with hamsters. Frontiers in neuroendocrinology.

[32] Rance NE, Dacks PA, Mittelman-Smith MA, Romanovsky AA, Krajewski-Hall SJ (2013). Modulation of body temperature and LH secretion by hypothalamic KNDy (kisspeptin, neurokinin B and dynorphin) neurons: a novel hypothesis on the mechanism of hot flushes. Frontiers in neuroendocrinology.

[33] Goodman RL, Coolen LM, Lehman MN (2014). A role for neurokinin B in pulsatile GnRH secretion in the ewe. Neuroendocrinology.

[34] Chung K (2013). Structural and molecular interrogation of intact biological systems. Nature.

[35] Susaki EA (2014). Whole-brain imaging with single-cell resolution using chemical cocktails and computational analysis. Cell.

[36] Lehman MN, Hileman SM, Goodman RL (2013). Neuroanatomy of the kisspeptin signaling system in mammals: comparative and developmental aspects. Advances in experimental medicine and biology.

[37] Clarke IJ, Cummins JT (1982). The temporal relationship between gonadotropin-releasing hormone (GnRH) and luteinizing hormone (LH) secretion in ovariectomized ewes. Endocrinology.

[38] Plant TM (2015). 60 Years of neuroendocrinology: The hypothalamo-pituitary–gonadal axis. Journal of Endocrinology.

[39] Renier N (2014). iDISCO: a simple, rapid method to immunolabel large tissue samples for volume imaging. Cell.

[40] Renier, N. *et al*. Mapping of Brain Activity by Automated Volume Analysis of Immediate Early Genes. *Cell* (2016). 10.1016/j.cell.2016.05.007 PMC4912438 27238021

[41] Erturk A (2012). Three-dimensional imaging of solvent-cleared organs using 3DISCO. Nature protocols.

[42] Belle M (2014). A simple method for 3D analysis of immunolabeled axonal tracts in a transparent nervous system. Cell reports.

[43] Soderblom, C. *et al*. 3D Imaging of Axons in Transparent Spinal Cords from Rodents and Nonhuman Primates. *eNeuro* **2**, 10.1523/eneuro.0001-15.2015 (2015). 10.1523/ENEURO.0001-15.2015 PMC4444235 26023683

[44] Belle M (2017). Tridimensional Visualization and Analysis of Early Human Development. Cell.

[45] Casoni, F. *et al*. Development of the neurons controlling fertility in humans: new insights from 3D imaging and transparent fetal brains. **143**, 3969–3981 (2016). 10.1242/dev.139444 27803058

[46] Desroziers E (2010). Mapping of Kisspeptin Fibres in the Brain of the Pro‐Oestrous Rat. Journal of neuroendocrinology.

[47] Kauffman AS, Navarro VM, Kim J, Clifton DK, Steiner RA (2009). Sex differences in the regulation of Kiss1/NKB neurons in juvenile mice: implications for the timing of puberty. American journal of physiology. Endocrinology and metabolism.

[48] Navarro VM (2011). Interactions between kisspeptin and neurokinin B in the control of GnRH secretion in the female rat. American Journal of Physiology-Endocrinology and Metabolism.

[49] Danzer SC, Price RO, McMullen NT, Rance NE (1999). Sex steroid modulation of neurokinin B gene expression in the arcuate nucleus of adult male rats. Brain research. Molecular brain research.

[50] Rance NE, Bruce TR (1994). Neurokinin B gene expression is increased in the arcuate nucleus of ovariectomized rats. Neuroendocrinology.

[51] Nestor CC (2012). Evidence of a role for kisspeptin and neurokinin B in puberty of female sheep. Endocrinology.

[52] Weems P (2017). Effects of Season and Estradiol on KNDy Neuron Peptides, Colocalization With D2 Dopamine Receptors, and Dopaminergic Inputs in the Ewe. Endocrinology.

[53] Yeo, S. H. *et al*. Visualisation of Kiss1 Neurone Distribution Using a Kiss1‐CRE Transgenic Mouse. *Journal of Neuroendocrinology* **28** (2016). 10.1111/jne.12435 PMC5091624 27663274

[54] Schwarz MK (2015). Fluorescent-protein stabilization and high-resolution imaging of cleared, intact mouse brains. PloS one.

[55] Stefaniuk M (2016). Light-sheet microscopy imaging of a whole cleared rat brain with Thy1-GFP transgene. Scientific reports.

[56] Overgaard A, Ruiz-Pino F, Castellano JM, Tena-Sempere M, Mikkelsen JD (2014). Disparate changes in kisspeptin and neurokinin B expression in the arcuate nucleus after sex steroid manipulation reveal differential regulation of the two KNDy peptides in rats. Endocrinology.

[57] Cheng G, Coolen LM, Padmanabhan V, Goodman RL, Lehman MN (2010). The Kisspeptin/Neurokinin B/Dynorphin (KNDy) Cell Population of the Arcuate Nucleus: Sex Differences and Effects of Prenatal Testosterone in Sheep. Endocrinology.

[58] McCosh RB (2017). Evidence That Endogenous Somatostatin Inhibits Episodic, but Not Surge, Secretion of LH in Female Sheep. Endocrinology.

[59] Lehman MN, Merkley CM, Coolen LM, Goodman RL (2010). Anatomy of the kisspeptin neural network in mammals. Brain Res.

[60] Lehman MN, Robinson JE, Karsch FJ, Silverman AJ (1986). Immunocytochemical localization of luteinizing hormone‐releasing hormone (LHRH) pathways in the sheep brain during anestrus and the mid‐luteal phase of the estrous cycle. Journal of Comparative Neurology.

[61] Lehman MN (1997). The GnRH system of seasonal breeders: anatomy and plasticity. Brain research bulletin.

[62] Becker K, Jahrling N, Saghafi S, Weiler R, Dodt HU (2012). Chemical clearing and dehydration of GFP expressing mouse brains. PloS one.

[63] Pan, C. *et al*. Shrinkage-mediated imaging of entire organs and organisms using uDISCO. **13**, 859–867, 10.1038/nmeth.3964 (2016). 10.1038/nmeth.3964 27548807

[64] Renier N (2016). Mapping of brain activity by automated volume analysis of immediate early genes. Cell.

[65] Strom, J. O., Theodorsson, A., Ingberg, E., Isaksson, I. M. & Theodorsson, E. Ovariectomy and 17beta-estradiol replacement in rats and mice: a visual demonstration. *Journal of visualized experiments: JoVE*, e4013, 10.3791/4013 (2012). 10.3791/4013 PMC3471296 22710371

[66] Steyn FJ, Anderson GM, Grattan DR (2007). Differential effects of centrally-administered oestrogen antagonist ICI-182,780 on oestrogen-sensitive functions in the hypothalamus. J Neuroendocrinol.

[67] Foradori CD, Goodman RL, Adams VL, Valent M, Lehman MN (2005). Progesterone increases dynorphin a concentrations in cerebrospinal fluid and preprodynorphin messenger ribonucleic acid levels in a subset of dynorphin neurons in the sheep. Endocrinology.

[68] Ciofi P, Leroy D, Tramu G (2006). Sexual dimorphism in the organization of the rat hypothalamic infundibular area. Neuroscience.

[69] Desroziers E (2010). Mapping of kisspeptin fibres in the brain of the pro-oestrous rat. J Neuroendocrinol.

