# Supplementary material for: Three-dimensional imaging of KNDy neurons in the mammalian brain using optical tissue clearing and multiple-label immunocytochemistry
Source: Sci Rep. 2018 Feb 2;8:2242. doi: 10.1038/s41598-018-20563-2 (PMC5797235; doi:10.1038/s41598-018-20563-2)
Supplement: Supplementary file 1 — Supplementary information [file 41598_2018_20563_MOESM1_ESM.pdf]

Supplementary Information

Three-dimensional imaging of KNDy neurons in the mammalian brain using optical tissue clearing and multiple-label immunocytochemistry

Aleisha M. Moore[1], Kathryn A. Lucas[1], Robert L. Goodman[3], Lique M. Coolen*[1,2] and Michael N. Lehman*[1]

[1]Dept. of Neurobiology & Anatomical Sciences, University of Mississippi Medical Center, Jackson, MS;

[2]Dept. of Physiology and Biophysics, University of Mississippi Medical Center, Jackson, MS;

[3]Dept. of Physiology and Pharmacology, West Virginia University, Morgantown, West Virginia.

*lcoolen@umc.edu, mlehman@umc.edu

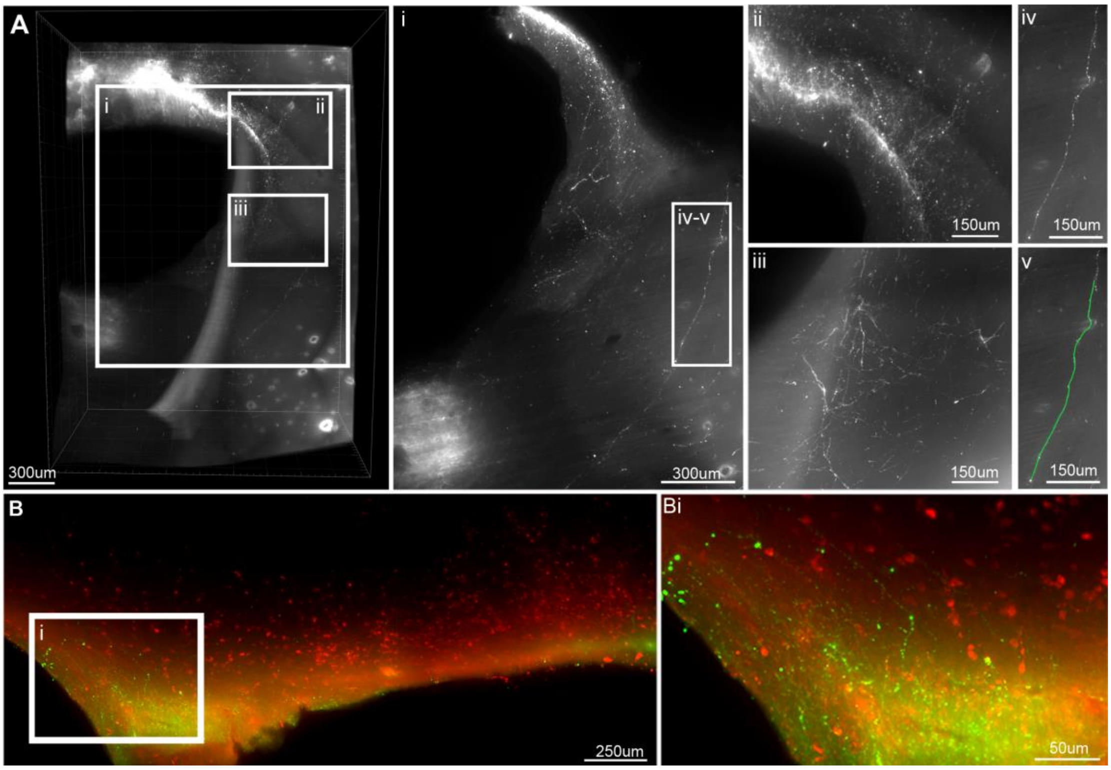

Supplementary Figure 1. GnRH and kisspeptin dual-label immunolabelling in the optically cleared ovine hypothalamus. A) 3D projection in the horizontal plane of GnRH immunolabelling in the median eminence and unilateral MBH/ARC of the ovine hypothalamus. i-iii) Projected images (100um optical thickness) of GnRH-ir at 100μm (i) and 800μm (ii-iii) from the most ventral point of imaging. High magnification view of a GnRH neuron axon in the MBH (iv) with neurolucida-style overlay (v). B) Sagittal projection of kisspeptin (red) and GnRH (green) immunolabelling in the ovine ARC (200μm optical thickness). i) Inset from B demonstrating kisspeptin cell bodies and GnRH fibers on the ventral surface of the arcuate nucleus.

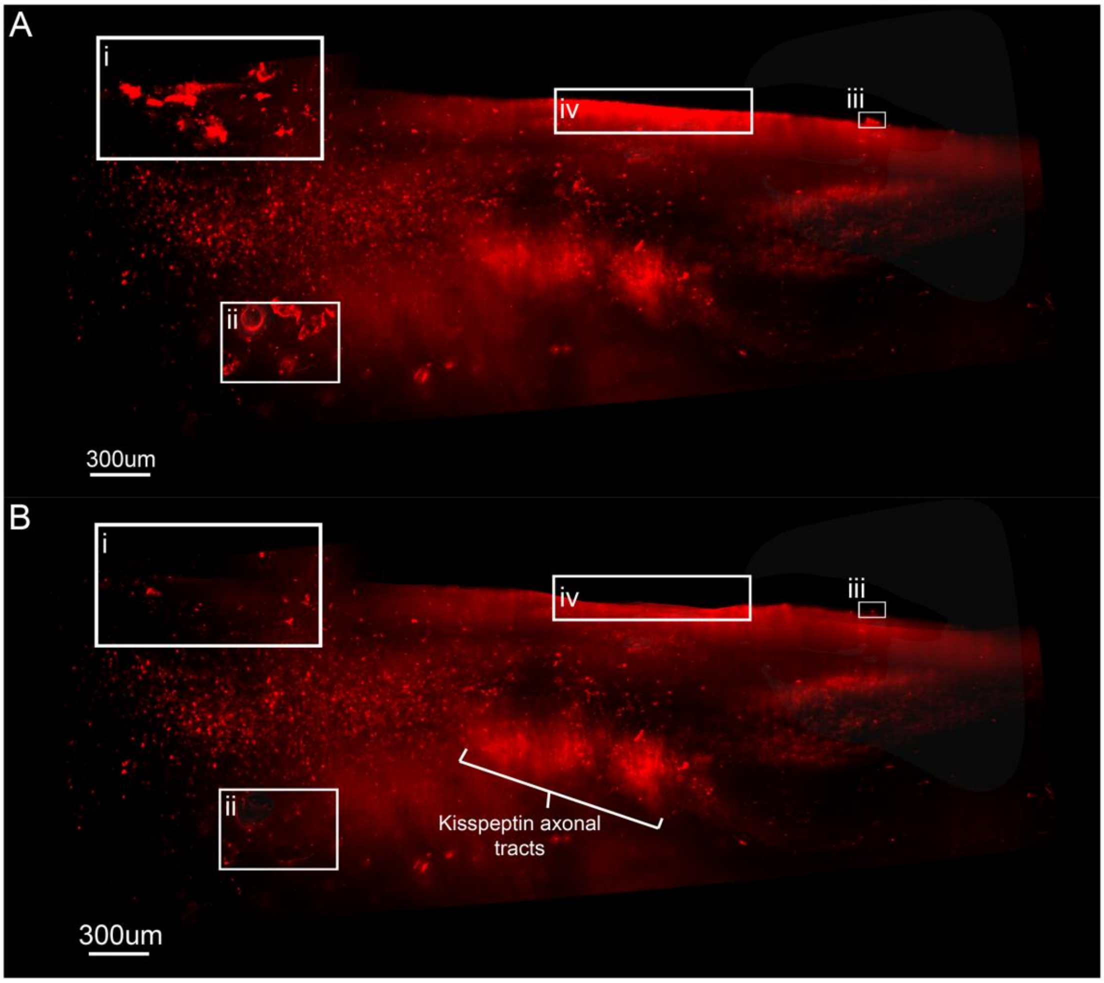

Supplementary Figure 2. 3D rendering in the horizontal plane of kisspeptin labelling in the sheep hypothalamus using IMARIS software. Before (A) and after (B) removal of large fluorescent blood vessels (i-iii) and a highly fluorescent portion of the third ventricle wall dorsal to the arcuate nucleus (iv).

Video 1. Animated 3D rendering of hypothalamic tyrosine hydroxylase neurons. 3D rendering using IMARIS software of tyrosine-hydroxylase-positive neurons within the periventricular nucleus of the third ventricle and paraventricular nucleus (dorsal to the optic chiasm and rostral to the arcuate nucleus).

Video 2. Animated 3D rendering of arcuate kisspeptin neurons in the ovine hypothalamus. 3D rendering using IMARIS software of kisspeptin immunolabelling within the unilateral ovine arcuate nucleus.
